# A Monoclonal Antibody ELISA–Based Assay for Measuring the Potency of Candidate H5 Clade 2.3.4.4b Pandemic Influenza Vaccines

**DOI:** 10.1111/irv.70206

**Published:** 2026-02-25

**Authors:** Marcus Odin, Falko Schmeisser, Jackeline Soto, Jerry P. Weir

**Affiliations:** ^1^ Laboratory of DNA Viruses, Division of Viral Products, Center for Biologics Evaluation and Research Food and Drug Administration Silver Spring Maryland USA

**Keywords:** H5, influenza, vaccine potency

## Abstract

**Background:**

The single radial immunodiffusion (SRID) assay is used to determine the potency of inactivated influenza vaccines. Nevertheless, development of alternative influenza vaccine potency assays with greater sensitivity and less reliance on large quantities of reference reagents is needed. Many candidate alternative potency assays use monoclonal antibodies (mAbs) to quantify the relevant immunogenic form of HA in vaccines. Although the feasibility of such assays has been demonstrated, concerns remain as to whether the necessary antibody reagents can be generated in the timeframes of vaccine manufacture, particularly for pandemic vaccines.

**Methods:**

We describe the development and evaluation of a mAb‐based ELISA assay to measure the potency of several candidate H5 clade 2.3.4.4b A/Astrakhan/3212/2020 influenza vaccines prepared recently in response to the ongoing H5N1 outbreak in the United States. The assay uses H5 mAbs that were generated and characterized several years prior to the emergence of these H5N1 viruses.

**Results:**

Correlation of the ELISA potency results with traditional SRID potency values was excellent for a cell‐based vaccine and one egg‐based vaccine. The ELISA and SRID potency values for the second egg‐based vaccine were not as well aligned initially, but alignment was improved by use of an internal standard.

**Conclusions:**

The results demonstrated that the ELISA potency assay is suitable for determining the potency of inactivated H5 A/Astrakhan/3212/2020 influenza vaccines. Moreover, the results demonstrate the importance of preparing libraries of antibody reagents for influenza subtypes with pandemic potential so that suitable mAbs are available for development of alternative mAb vaccine potency assays when needed.

## 1 Introduction

The single radial immunodiffusion (SRID) assay has been the standard method for measuring the potency of inactivated influenza vaccines since 1978 and is also now used for measuring the potency of recombinant vaccines [1]. The agarose gel‐based assay uses an influenza strain‐specific antibody preparation to quantify the influenza hemagglutinin (HA) in vaccine samples by comparison to the assigned HA value of a reference antigen standard [2]. There are many practical attributes of the SRID for use as a potency assay, including simplicity, reproducibility, and the correlation of potency values to clinical relevance. Nevertheless, alternative potency assays are desirable to provide improved sensitivity, to reduce the quantity of assay reagents needed by vaccine manufacturers, and to provide assay flexibility for manufacturing, particularly in the event of an influenza emergency when timelines for vaccine manufacture and release would be compressed.

Considerable effort has been expended in developing and evaluating alternative potency assays for HA‐based influenza vaccines, and a number of potential alternative assays have shown feasibility in early stages of development. Many candidate potency assays rely on the use of monoclonal antibodies (mAbs) to bind and quantify the relevant immunogenic form of HA in vaccine samples; examples of such antibody‐based assays include several types of ELISA [3, 4, 5, 6], antibody printed slides [7, 8], and antibody‐based surface plasmon resonance [9] or biolayer interferometry [10] assays. A number of these assays such as ELISAs and antibody printed slides are amenable to automation and high throughput. Critical aspects of all such mAb‐based assays include the identification, selection, and availability of appropriate antibodies for assay set‐up, and the question of whether such antibody reagents could be generated in the timeframe required for vaccine manufacture, especially for a vaccine response to an emerging influenza pandemic. We have previously suggested that one approach to addressing this latter concern would be the advanced preparation of well characterized panels of mAbs for influenza subtypes with pandemic potential, in order to increase the likelihood of having mAbs available for development of an alternative mAb potency assay if needed [10].

The appearance and rapid spread of highly pathogenic avian influenza (HPAI) H5 viruses in North America and the United States beginning in 2021, producing large outbreaks in wild aquatic birds, commercial poultry, and dairy cows, and some human infections [11, 12, 13, 14], prompted the US Department of Health and Human Services (HHS) to contract for the production of pilot lots of H5 vaccine for clinical evaluation. The availability of several H5N8 A/Astrakhan/3212/2020 candidate vaccines allowed us to test the premise that previously prepared H5 mAbs [15] might be acceptable for development of an alternative vaccine potency assay. In the study described here, we describe an H5 ELISA‐based assay for measuring the potency of candidate H5 clade 2.3.4.4b pandemic influenza vaccines in comparison to the traditional SRID potency assay for these vaccines. The results indicate that H5 mAbs prepared several years prior to the emergence of H5 clade 2.3.4.4b viruses in the United States are suitable for use in an alternative potency assay for H5 influenza A/Astrakhan/3212/2020 vaccines and emphasize the importance of developing, updating, and maintaining panels of mAbs for influenza subtypes with pandemic potential.

## 2 Results

### 2.1 SRID Potency of A/Astrakhan/3212/2020 Vaccines

**FIGURE 1:**
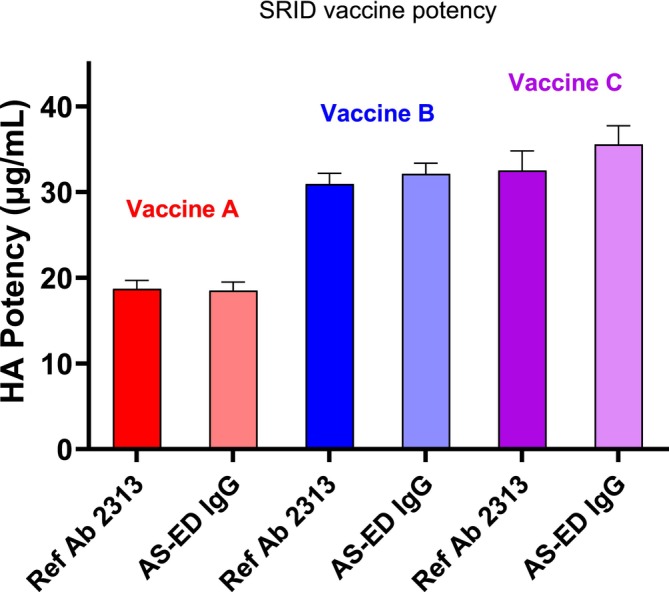
SRID potency for three A/Astrakhan/3212/2020 vaccines. The potency and standard deviation of A/Astrakhan/3212/2020 (H5N8) vaccines from three manufacturers was determined by SRID analysis using either the standard reference antisera (Ref Ab 2313) prepared by traditional methods or an alternative antibody IgG prepared using a recombinant H5 hemagglutinin as the immunogen (AS‐ED IgG). Vaccine A is a cell‐based inactivated vaccine formulated at a nominal 15 μg/mL. Vaccines B and C are egg‐based inactivated vaccines formulated at a nominal 30 μg/mL.

In an earlier study, we measured the potency of a cell‐based (Vaccine A) and an egg‐based H5 A/Astrakhan/3212/2020 vaccine (Vaccine B) in the established SRID potency assay using the standard reference antibody prepared by traditional methods in comparison to an alternative antibody prepared using a recombinant H5 hemagglutinin (rHA) as immunogen [16]. SRID assays on these vaccines, as well as on a 2nd egg‐based vaccine (Vaccine C), were performed in the current study to ascertain whether there had been any decrease in SRID vaccine potency since the earlier study and to provide a contemporaneous comparison to results derived from an alternative antibody‐capture ELISA potency assay. Because we intended to utilize the alternative antibody reagent described in the previous study in the antibody‐capture ELISA potency assay, SRID assays were conducted with both the standard potency antiserum used for SRID reagent calibration and the purified IgG A/Astrakhan/3212/2020 alternative potency antibody reagent. The results of the SRID assays for the three vaccines using the standard and the alternative potency antibody reagents are shown in Figure 1. For each vaccine, there was no statistical difference between the potency measured using the standard antibody reagent (Ab 2313) compared to the alternative potency antibody reagent prepared using the Astrakhan ectodomain (ASED IgG), and SRID values of 18.8, 31.1, and 34.0 μg/mL were assigned for Vaccines A, B, and C, respectively. The SRID values obtained here for Vaccines A and B were similar to those previously reported [16].

### 2.2 Development of a mAb Capture ELISA Potency Assay for a Cell‐Based H5 Influenza A/Astrakhan/3212/2020 Vaccine

**FIGURE 2:**
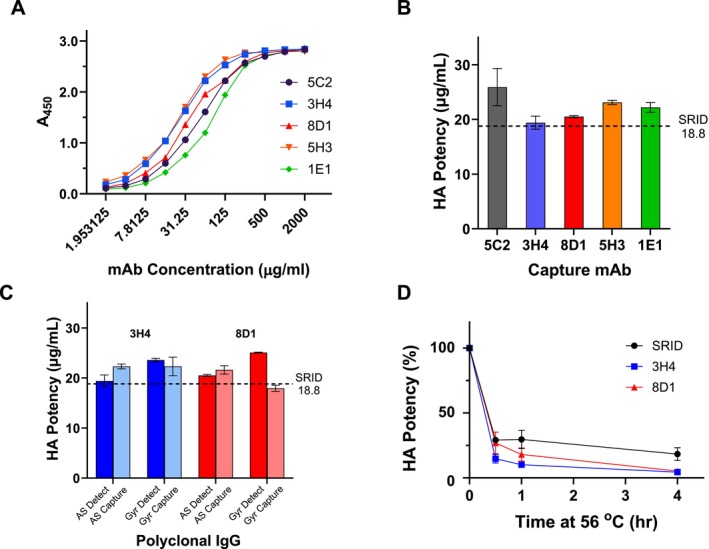
ELISA potency assay for cell‐based A/Astrakhan/3212/2020 vaccine. (A) mAb binding to the cell‐based A/Astrakhan/3212/2020 Reference Antigen CBER H5‐Ag‐2213. Plates were coated with Reference Antigen 2213 at 1 μg/mL. Each mAb was twofold serially diluted from an initial concentration of 2 mg/mL mAb. Horseradish peroxidase labeled goat antimouse antibody was used for detection and the binding measured by absorbance (A_450_). (B) Potency and standard deviation of a cell‐based A/Astrakhan/3212/2020 vaccine was determined by mAb‐capture ELISA using the indicated murine mAbs. (C) Potency and standard deviation of a cell‐based A/Astrakhan/3212/2020 vaccine was determined by antibody‐capture ELISA using a polyclonal IgG for detection following mAb capture (dark bars) or polyclonal IgG for capture with mAb for detection (lighter bars). Polyclonal IgG was prepared to either A/Astrakhan/3212/2020 (AS) or A/gyrfalcon/Washington/41088‐6/2014 (Gyr). (D) Potency of stressed cell‐based A/Astrakhan/3212/2020 vaccine. Vaccine was treated at 56°C for 0.5, 1, or 4 h and then tested by SRID or ELISA potency. The residual HA potency at each timepoint for each assay is shown as a percentage of the starting potency value.

We previously described a panel of mAbs made to an H5 clade 2.3.4.4c A/gyrfalcon/Washington/41088‐6/2014 virus hemagglutinin [15]. In that study, we found that all of the 2.3.4.4c mAbs bound to and neutralized other tested 2.3.4.4 subclade viruses, including a 2.3.4.4b A/Astrakhan/3212/2020 virus whose hemagglutinin (HA) is closely related to the HA of highly pathogenic avian influenza (HPAI) 2.3.4.4b viruses currently causing large outbreaks in wild aquatic birds, commercial poultry, and dairy cows in the United States [11, 13]. To explore whether these mAbs could be used to develop an ELISA vaccine potency assay, we tested these 2.3.4.4c HA mAbs for their ability to bind the A/Astrakhan/3212/2020 Reference Antigen that was recently developed and calibrated for use in the SRID vaccine potency assay for cell‐based vaccines (Figure 2A). We also included a cross‐reactive H5 mAb, designated as 5C2, that was previously shown to neutralize A/Astrakhan 3212/2020. All of the tested H5 mAbs bound well to the cell‐based reference antigen, suggesting that they might be suitable for use in developing an antibody‐capture ELISA potency assay for A/Astrakhan/3212/2020 vaccines, similar to assay methodology described previously [5, 10].

Although ELISA assays can be set up in various formats, we have previously used mouse mAbs as the capture antibodies and a rabbit polyclonal antibody reagent for detection. The ELISA potency values are determined by comparing the binding of HA in vaccine samples relative to the binding of HA in a reference antigen standard that has an assigned value in μg of HA. Using this assay format for the cell‐based Vaccine A, formulated at a nominal 15 μg/mL concentration, we evaluated the ability of the H5 mAbs to serve as capture antibodies (Figure 2B). All of the tested mAbs were able to capture reference antigen and quantify the HA in Vaccine A, but the potency values of 19.4 ± 1.2 and 20.5 ± 0.2 obtained with 3H4 and 8D1, respectively, were within 10% of the SRID determined potency value of 18.8 μg/mL (Figure 2B) and were closer to the SRID value than the potency values determined with the other capture mAbs. Assays using the cross‐reactive H5 mAb 5C2 yielded the most discordant potency results compared to the SRID potency values. As a result of these assay results, H5 mAbs 3H4 and 8D1 were selected for further assay development and evaluation.

We also determined the potency of Vaccine A by varying other parameters in the ELISA assay, including evaluating a polyclonal antibody made to the older H5 clade 2.3.4.4c A/gyrfalcon/Washington/41088‐6/2014 HA and an ELISA format that used polyclonal antibody for capture with either 3H4 or 8D1 as the detection antibody. As shown in Figure 2C, assays using 3H4 or 8D1 as capture and the A/gyrfalcon polyclonal as detection produced somewhat higher potency values than those obtained with the A/Astrakhan polyclonal antibody. Assays' set‐up using a polyclonal antibody as capture and 3H4 or 8D1 as the detection antibody also generated reasonably acceptable potency values, all within 20% of the SRID value and one combination (A/gyrfalcon polyclonal capture—8D1 detection) within 5% of the SRID value.

Finally, we assessed the ability of the 3H4 and 8D1 to detect loss of potency due to stress. Heat treatment of the cell‐based H5 influenza A/Astrakhan vaccine at 56°C led to a rapid loss of SRID potency (Figure 2D). In fact, accurate quantification of the heat‐treated samples by SRID at all time points was difficult because the assay is not particularly sensitive for low HA concentrations. ELISA potency assays with both 3H4 and 8D1 were capable of quantifying the reduced potency in the heat‐treated samples and showed a continuing drop in potency with length of heat exposure, confirming that the mAb capture ELISA is sensitive to loss of HA integrity.

Taken together, the results indicate that a mAb‐capture ELISA using the H5 mAbs 3H4 and 8D1, with polyclonal rabbit AS‐ED IgG as the detection Ab, is an acceptable alternative potency for cell‐based H5 influenza A/Astrakhan vaccines, but the results also suggest that there is flexibility in the design and format of the assay.

### 2.3 Development of a mAb Capture ELISA Potency Assay for Egg‐Based H5 Influenza A/Astrakhan/3212/2020 Vaccines

**FIGURE 3:**
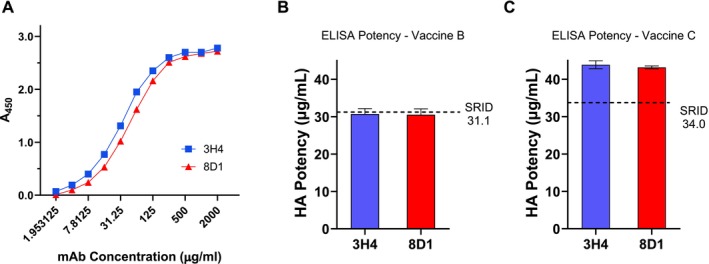
ELISA potency assay for egg‐based A/Astrakhan/3212/2020 vaccines. (A) mAb binding to the egg‐based A/Astrakhan/3212/2020 Reference Antigen CBER H5‐Ag‐2301. Plates were coated with Reference Antigen 2301 at 1 μg/mL. Each mAb was twofold serially diluted from an initial concentration of 2 mg/mL mAb. An HRP‐labeled goat antimouse antibody was used for detection and the binding measured by absorbance (A_450_). (B) Potency and standard deviation of egg‐based A/Astrakhan/3212/2020 Vaccine B was determined by mAb‐capture ELISA using mAbs 3H4 and 8D1. (C) Potency and standard deviation of egg‐based A/Astrakhan/3212/2020 Vaccine C were determined by mAb‐capture ELISA using mAbs 3H4 and 8D1.

In further investigations, we evaluated H5 mAbs 3H4 and 8D1 in an antibody capture ELISA to determine the potency of egg‐based A/Astrakhan vaccines. Both mAbs bound well to the egg‐based Reference Antigen (Figure 3A), and we used them in our basic assay format, capturing reference antigen and vaccine with mAbs and detection with the polyclonal A/Astrakhan IgG to determine the potency of egg‐based A/Astrakhan vaccines from two manufacturers. Both of these vaccines were formulated at a nominal 30 μg/mL concentration. There was good concordance between the ELISA potency results using either capture mAb and the SRID potency results for Vaccine B (Figure 3B), with the ELISA potency values within 2% of the SRID potency value, indicating that the mAb‐capture ELISA was an acceptable alternative potency assay for this vaccine. The ELISA and SRID potency values for Vaccine C were not as well aligned (Figure 3C), differing by about 30%, suggesting assay modification or additional assay parameter optimization might be useful for this vaccine.

**FIGURE 4:**
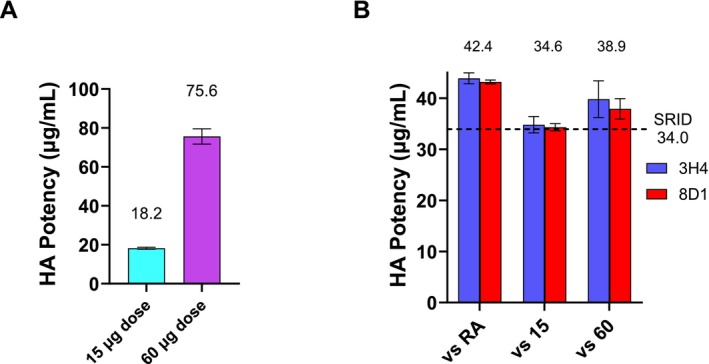
ELISA potency assay for egg‐based A/Astrakhan/3212/2020 Vaccine C using an alternative reference reagent. (A) The potency and standard deviation of nominal 15 and 60 μg/mL formulations of egg‐based A/Astrakhan/3212/2020 (H5N8) Vaccine C were determined by SRID analysis using the standard reference antisera (Ref Ab 2313). (B) Potency and standard deviation of the 30 μg/mL formulation of egg‐based A/Astrakhan/3212/2020 Vaccine C were determined by mAb‐capture ELISA using mAbs 3H4 and 8D1 and either the calibrated reference Antigen CBER H5‐Ag‐2301 or the 15 and 60 μg/mL formulations of vaccine as a reference.

One alternative to extensive assay optimization, and the time inherent in that process, is the use of a manufacturer‐specific internal standard that is directly calibrated to the reference antigen and then used for subsequent vaccine analysis. Although we did not have such a standard for Vaccine C, we were able to obtain two additional Vaccine C formulations, one at a nominal 15 μg/mL and another at a nominal 60 μg/mL, and we investigated the use of these samples as substitutes for a Vaccine C internal standard for the 30 μg/mL formulation. SRID analysis established values of 18.2 and 75.6 for the 15 and 60 μg/mL Vaccine C formulations, respectively (Figure 4A). When the 60 μg/mL formulation was set to its SRID value and used as an internal reference, the ELISA potency value of the 30 μg/mL Vaccine C differed by less than 15% of the SRID determined potency. Similarly, when the 15 μg/mL vaccine was set to its SRID value and used as an internal reference, the ELISA potency value of the 30 μg/mL Vaccine C was within 2% of the SRID value. These results suggest that by establishing an internal reference standard linked to the SRID, the mAb capture ELISA can be used for potency determination of an H5 egg‐based vaccine even when initial results are not acceptably aligned.

## 3 Discussion

Following the 2009 H1N1 pandemic, efforts to develop alternative potency assays for influenza vaccines intensified. This was driven by the fact that reagent preparation for the traditional SRID potency assay is time‐consuming and the amounts of reagents needed by manufacturers and regulatory authorities are immense. In fact, during the 2009 H1N1 pandemic, alternative methods were used in several instances to quantify vaccine potency for use in clinical trials because reagents were not available in sufficient quantities [17]. In the ensuing years, numerous assays have been developed and evaluated as potential alternatives to the SRID by various manufacturers, regulators, and research institutes, and there has been a great deal of collaboration among these influenza vaccine stakeholders to move these efforts forward, in particular to enhance pandemic preparedness [18].

Antibody‐based and non‐antibody–based alternative influenza vaccine potency assays have been reported. Non‐antibody–based assays include physicochemical methods such as high‐pressure liquid chromatography [19, 20], isotope dilution mass spectroscopy [21], and surface plasmon resonance [22]. Although such methods are sensitive and accurate, by themselves, they generally lack the ability to quantify only the conformationally and antigenically correct HA in a sample. Modifications that incorporate either HA receptor binding, HA antibody binding, or limited protease digestion have mitigated that assay limitation for some of these techniques [9, 23, 24, 25, 26, 27]. Antibody‐based methods are attractive as alternative vaccine potency assays because they are capable of distinguishing and quantifying the relevant immunogenic form of HA in vaccine samples, and several examples of such candidate assays have been developed and described [3, 5, 6, 7, 9, 10, 28]. The key issues that have to be addressed in any antibody‐based assay are the selection criteria of appropriate antibodies and the availability of the antibodies. Selection is still largely empirical, but screening by binding and functional attributes such as neutralization and hemagglutination inhibition can be performed fairly rapidly. Nevertheless, all mAbs with similar attributes do not always perform similarly in an assay, so a large diverse panel of mAbs is important to enable successful assay development. Because of the time involved in developing and characterizing a panel of mAbs, it is not practical to begin this process only when a decision has been made to initiate vaccine production. The feasibility of an alternative potency assay for a pandemic vaccine response would depend on the availability of preexisting mAbs that were suitable for use in the assay.

The goal of the study reported here was to explore whether a group of H5 mAbs, made to the HA of the H5N8 virus A/gyrfalcon/Washington/41088‐6/2014 (clade 2.3.4.4c), could be used to develop an ELISA potency assay for A/Astrakhan/3212/2020 (clade 2.3.4.4b) vaccines. We also evaluated mAb 5C2, a mAb made to the HA of the clade 2.3.2.1a virus H5 A/duck/Bangladesh/19097/2013 that was broadly neutralizing for almost all H5 viruses tested to date. All four of the 2.3.4.4c mAbs and the cross‐neutralizing 2.3.2.1a mAb were able to capture and quantify HA in the potency ELISA, but there were differences in how well the potency results generated with the individual mAbs compared to the SRID values. The cross‐reactive 5C2 ELISA potency results for the cell‐based vaccine were nearly 40% higher than the corresponding SRID value, suggesting that 5C2 bound and captured the detergent‐split vaccine sample more strongly than the inactivated reference antigen. Although a detailed map of the 5C2 binding epitope is not known, results from escape mutant mapping studies indicated that 5C2 does not bind the globular head of HA but rather somewhere between the globular head and stalk region. This likely explains the lack of hemagglutination inhibition and the extensive cross‐reactivity with H5 influenza viruses [15] and may explain in part how detergent treatment may result in augmented binding and capture of vaccine relative to a reference antigen.

The H5 2.3.4.4c mAbs 3H4, 8D1, 5H3, and 1E1 are all derived from the same A/gyrfalcon/Washington/41088‐6/2014 mAb panel and, though they are distinct mAbs, all compete for binding of the same HA Sa antigenic site [15]. ELISA potency results using 3H4 and 8D1 were closest to the assigned SRID potency value for cell‐based Vaccine A and were chosen for extended analyses. Overall, results obtained with these two mAbs and all three vaccines were very similar and not significantly different, suggesting that the assay results using both mAbs could be combined if needed. A limited amount of exploratory assay work was done using a polyclonal antibody for capture combined with a mAb for detection and also the use of the heterologous A/gyrfalcon/Washington/41088‐6/2014 polyclonal antibody in the assay. Although none of these permutations showed an obvious advantage over our standard assay format, it seems very likely that any of these assay formats and reagents could be made acceptable with additional optimization.

The ELISA potency assay results for Vaccines A and B corresponded well with their relative SRID potency values, with differences of less than 10%, indicating that the mAb‐capture ELISA assays described here could be used for potency determination of these vaccines with little or no additional optimization. The divergence between the capture ELISA results and the SRID potency value for Vaccine C was approximately 30%. Although this difference may be acceptable under some circumstances, the results suggested that additional optimization is warranted for this vaccine. It is not clear why the initial ELISA results differed from the SRID results by 30% for Vaccine C but were extremely well aligned for vaccine A and B (< 10%). Interestingly, the calibrated cell‐ and egg‐based reference antigens were produced by the manufacturers of Vaccines A and B, respectively. It is possible that there are subtle differences in virus expansion, inactivation, and manufacturing between egg‐based Vaccines B and C that might account for differences in the way that mAbs interact with a reference antigen produced by one manufacturer and the vaccine produced by another manufacturer. Nevertheless, a discrepancy of only 30% between the ELISA potency result and the SRID value should be amenable to mitigation with further assay optimization. Using other vaccine formulations as a model, we demonstrated the possibility that a manufacturer‐specific internal standard, directly calibrated to the reference antigen, might be another acceptable alternative.

Overall, there are several key takeaways from the present study. First, the described mAb‐capture ELISA is an acceptable potency assay, with minimal or modest additional optimization needed for candidate inactivated A/Astrakhan/3212/2020 vaccines that have been manufactured for preparedness purposes in response to the H5N1 clade 2.3.4.4b virus outbreak in the United States. Second, the mAbs used in the assays were already available prior to the emergence of the currently circulating 2.3.4.4b viruses, providing additional support for the premise that mAb availability may not be a limiting factor for ELISA potency assays if a sufficiently large and diverse panel of influenza mAbs is maintained. Third, the polyclonal antibodies used in the assay in this study can be prepared in advance as part of a reagent library both for SRID and ELISA potency assays. Fourth, neither the mAbs nor the polyclonal antibodies used in the ELISA assay required the availability of live influenza virus. Fifth, because the use of an ELISA as a potency assay for a pandemic influenza vaccine reduces the amount of calibrated reagents needed by manufacturers compared to the SRID, it is probable that smaller lots of reagents would suffice for a manufacturing campaign. Also, if traditional SRID reagents were not available at an early stage of an avian influenza outbreak, alternative potency assays would be valuable to provide an approximation of HA content to facilitate initiation of manufacturing.

Taken together, the results of the current study are encouraging for development of alternative potency assays for pandemic influenza vaccines. Nevertheless, additional work is needed before such alternative assays are feasible for implementation even in an emergency situation. As already noted, the selection criteria for mAb reagents need further defining. Mechanisms also need to be put into place to ensure timely updating of such antibody reagent panels and to produce the quantities of antibody reagents needed to support vaccine production. Although we were fortunate to have three different H5N8 vaccines available for evaluation, more extensive testing of vaccines from more manufacturers and of additional subtypes would strengthen confidence in any alternative methodology and would be particularly important for use of an alternative methodology without prior validation against the SRID. Finally, although not addressed in this study, the development of an alternative method for producing a reference antigen that is independent of the vaccine manufacturing process and could be used by all types of inactivated vaccines (e.g., egg‐based, cell‐based, and recombinant) would be an advantageous improvement to expedite potency assay development and manufacturing for pandemic influenza vaccines.

## 4 Materials and Methods

### 4.1 Vaccines

Inactivated influenza vaccines prepared to the H5N8 candidate vaccine virus A/Astrakhan/3212/2020 were produced by licensed US vaccine manufacturers through contracts with the US Department of Health and Human Services (HHS), Administration for Strategic Preparedness and Response (ASPR), and Biomedical Advanced Research and Development Authority (BARDA).

### 4.2 Influenza H5 Monoclonal Antibodies

Murine mAbs to the hemagglutinins of H5 clade 2.3.2.1 H5N1 A/duck/Bangladesh/19097/2013 and clade 2.3.4.4c H5N8 A/gyrfalcon/Washington/41088‐6/2014 were previously described [15].

### 4.3 Influenza H5 Polyclonal Antibodies

The polyclonal IgG antibody to the ectodomain of H5N8 A/Astrakhan/3212/2020 or A/gyrfalcon/Washington/41088‐6/2014 was prepared as previously described [16]. CBER H5‐Ab2313 antiserum reagent was prepared by immunization of sheep with bromelain cleaved HA from egg‐grown A/Astrakhan/3212/2020.

### 4.4 Measurement of Potency by Single Radial Immunodiffusion (SRID)

The SRID assay is based on the diffusion of detergent‐disrupted virus or antigen into an agarose gel containing antigen‐specific influenza HA antibodies and was performed as previously described [10, 29]. The vaccine potency was determined by the parallel line bioassay method using reference and test‐vaccine dose–response curves (log antigen dilution vs. log zone diameter). Replicates were included in each SRID assay, and three to seven individual assays were performed for each antigen–antibody combination to determine the mean value and standard deviation. The following reference reagents were used:

1. CBER H5‐Ag‐2213 Reference Antigen—a lyophilized preparation of purified BPL inactivated H5N8 A/Astrakhan/3212/2020 virus grown in MDCK cells. HA concentration—98 μg/mL.
2. CBER H5‐Ag‐2301 Reference Antigen—a lyophilized preparation of purified formalin inactivated H5N8 A/Astrakhan/3212/2020 virus grown in eggs. HA concentration—62 μg/mL.

### 4.5 Measurement of Potency by Antibody ELISA

#### 4.5.1 mAb Capture‐Polyclonal Detection

One‐hundred microliters per well of murine mAbs were used to coat 96‐well Immulon 2HB flat bottom microtiter plates (ThermoFisher Scientific) at a concentration of 1 μg/mL for the cell‐based vaccine and 2 μg/mL for the egg‐based vaccines in coating solution (SeraCare) overnight at 4°C. The following day, plates were blocked with 200 μL/well of PBS/10% fetal bovine serum (FBS) for 1 h at 37°C. In parallel, reference antigen and vaccines were diluted 1:10 and treated with 1% Zwittergent 3‐14 (Millipore Sigma) for 30 min at room temperature and then diluted with PBS/0.05% Tween/10% FBS to experimentally determined concentrations. Following incubation, plates were washed five times with 300 μL per well per wash of PBS/0.05% Tween/10% FBS (BioTek 405 TS microplate washer, Agilent BioTek). Samples were serially diluted in PBS/0.05% Tween/10% FBS followed by incubation at 37°C for 2 h. Plates were washed five times, and 100 μL/well of influenza H5 polyclonal antibody at 1:2000 was added before incubating at 37°C for 1.5 h. Plates were washed again five times, and 100 μL per well of horseradish peroxidase labeled goat antirabbit IgG (SeraCare) at 1:2000 in PBS/0.05% Tween/10% FBS was added followed by incubation at 37°C for 1 h. Plates were washed seven times, and 100 μL per well of ABTS peroxidase (SeraCare) was added and allowed to react for 30 min before stopping the reaction by adding an additional 100 μL per well of ABTS peroxidase stop solution (SeraCare). HA concentration was determined by parallel line analysis of the four‐parameter regression fits of test vaccine samples to the reference antigen standard using a VersaMax Microplate Reader and SoftMax Pro 7.0 (Molecular Devices). The mean and standard deviation were determined from four to five individual experiments.

#### 4.5.2 Polyclonal Antibody Capture‐mAb Detection

The assay procedure was similar to that described above except that 100 μL per well of H5 polyclonal antibodies was used to coat 96‐well Immulon 2HB flat bottom microtiter plates at a concentration of 5 μg/mL in coating solution. For detection, murine mAbs were used at a concentration of ~0.57 μg/mL (100 μL per well), and horseradish peroxidase labeled goat antimouse IgG (SeraCare) was used at 1:2000. The mean and standard deviation were determined from two to four individual experiments.

### 4.6 Statistical Analysis

Differences in potency values between tested vaccines were compared using a statistical *t* test, and a *p* value of < 0.05 was considered statistically significant. Statistical tests were performed using GraphPad Prism Version 9.0.0 for Windows (GraphPad Software, San Diego, California, United States; www.graphpad.com).

## Author Contributions

J.P.W. designed the study. M.O., F.S., and J.S. prepared reagents and performed experiments. M.O., F.S., J.S., and J.P.W. analyzed and interpreted data and prepared figures. M.O. and J.P.W. wrote the manuscript with input, review, and concurrence of all authors.

## Funding

The research presented in this manuscript was supported in part by the US Department of Health and Human Services (HHS), Administration for Strategic Preparedness and Response (ASPR), and Biomedical Advanced Research and Development Authority (BARDA) under Contract Numbers 75A50122D00002 and 75A50122D00004.

## Conflicts of Interest

The authors declare no conflicts of interest.

## Data Availability

The authors declare that all relevant data supporting the findings of this study are available within the paper. Reagents generated in the study are available upon request.
